# Factors associated with health-related quality of life among community-dwelling older adults: the APPCARE study

**DOI:** 10.1038/s41598-024-64539-x

**Published:** 2024-06-21

**Authors:** Esmée L. S. Bally, Sophie A. Korenhof, Lizhen Ye, Amy van Grieken, Siok Swan Tan, Francesco Mattace-Raso, Elena Procaccini, Tamara Alhambra-Borrás, Hein Raat

**Affiliations:** 1 Department of Public Health, Erasmus MC University Medical Center, Rotterdam, The Netherlands; 2 Research Group City Dynamics, InHolland University of Applied Sciences, Rotterdam, The Netherlands; 3 Division of Geriatric Medicine, Erasmus MC University Medical Center, Rotterdam, The Netherlands; 4 Funded Project Office, Local Health Authority n.2 Treviso, Treviso, Italy; 5 Polibienestar Research Institute, University of Valencia, Valencia, Spain

**Keywords:** Environmental social sciences, Health care, Medical research, Risk factors

## Abstract

This study aimed to identify the factors associated with health-related quality of life (HRQOL) among community-dwelling older adults. Physical and mental HRQOL were measured by the 12-item Short Form Health Survey (SF-12) at baseline and follow-up. Linear regression models were used to evaluate associations between socio-demographic, health, and lifestyle factors and HRQOL. The sample included 661 participants (mean age = 77.4 years). Frailty was negatively associated with physical HRQOL (B = − 5.56; *P* < 0.001) and mental HRQOL (B = − 6.65; *P* < 0.001). Participants with a higher score on activities of daily living (ADL) limitations had lower physical HRQOL (B = − 0.63; *P* < 0.001) and mental HRQOL (B = − 0.18; *P* = 0.001). Female sex (B = − 2.38; *P* < 0.001), multi-morbidity (B = − 2.59; *P* = 0.001), and a high risk of medication-related problems (B = − 2.84; *P* < 0.001) were associated with lower physical HRQOL, and loneliness (B = − 3.64;* P* < 0.001) with lower mental HRQOL. In contrast, higher age (B = 2.07; *P* = 0.011) and living alone (B = 3.43; *P* < 0.001) were associated with better mental HRQOL in the multivariate models. Future interventions could be tailored to subpopulations with relatively poor self-reported HRQOL, such as frail or lonely older adults to improve their HRQOL.

## Introduction

In the European Union (EU), the proportion of people aged 65 and older is expected to rise substantially, from 20.6% in 2020 to 29.4% in 2050^1^. This demographic change is primarily driven by historically low birth rates and an increased life expectancy^2^. Across the EU in 2018, men and women aged 65 years had an average life expectancy of 18.1 and 21.6 years respectively^1^. However, at age 65 years, both men and women spend approximately half of their remaining lives with limitations in functioning^1^. Chronic conditions such as diabetes, osteoporosis, cardiovascular disease, and dementia are increasingly common among older adults^3^. These conditions may negatively impact an older person’s functional independence and quality of life^4^.

The World Health Organization defines quality of life as ‘an individual’s perception of their position in life in the context of the culture and value systems in which they live and in relation to their goals, expectations, standards and concerns’^5^. Health-related quality of life (HRQOL) comprises those aspects of quality of life that relate to a person’s perception of health^6^. It is a key patient-reported outcome and usually includes various domains of health, such as general health, physical functioning, mental health, social functioning and role function^7^. HRQOL can be used to assess the impact of disease on a person’s life as well as within the general population^6^. An example of a generic scale that has been developed to measure HRQOL is the 12-item Short Form Health Survey (SF-12)^8^. The SF-12 includes eight scales yielding two summary measures: physical and mental health.

Measuring HRQOL has become an important component of public health surveillance and can be considered a valid indicator of unmet needs and intervention outcomes^6^. HRQOL data analysis supports the identification of subgroups with relatively poor self-reported health. Interpretation and publication of these data can help to allocate resources more efficiently and to monitor the effectiveness of community interventions^5^. Previous studies have identified associations between HRQOL and socio-demographic factors, including sex and lower education^9,10^. Furthermore, chronic conditions, frailty, low levels of physical activity, and lack of social support have been associated with poor self-reported HRQOL^10–13^.

Thus far, studies have shown mixed results concerning the factors associated with HRQOL. Most studies have focused on HRQOL in relation to specific diseases or subpopulations. There is a need for a comprehensive view by studying the factors associated with HRQOL in the general population. New insights into the relationships between HRQOL and risk factors (e.g. socio-demographic factors, health-related factors, and lifestyle factors) can improve tailoring interventions to subpopulations with poor self-reported health, to improve their situation and avert more severe consequences. This study aims to identify the factors associated with HRQOL among community-dwelling older adults.

## Methods

### Study design

The present study used baseline and follow-up data from the ‘Appropriate care paths for frail elderly people: a comprehensive model’ (APPCARE) study—a prospective cohort study funded by the European Commission, under Grant Agreement number 664689. The APPCARE study aimed to promote healthy ageing among older adults. The project has been conducted in three European sites (Rotterdam, the Netherlands; Treviso, Italy; and, Valencia, Spain). The current study used baseline and 6-months follow-up data from the Rotterdam site.

### Study participants

In collaboration with the Municipality of Rotterdam, 865 community-dwelling older adults (≥ 65 years) were invited by letter to participate in the study. Participants’ eligibility for the study was assessed by an employee of the Municipality of Rotterdam by screening the Municipal Personal Records Database. The inclusion criteria were as follows: (1) living in the municipality of Rotterdam; (2) age 65 years or older, (3) community-dwelling (not in long-term care) at the time of recruitment, and (4) able to provide written informed consent to participate in the study. An information package, including an information sheet, informed consent form, baseline questionnaire, and prepaid envelope was sent by post to eligible citizens. Participants who returned the signed informed consent and filled in the baseline questionnaire were included in the study. After 6 months, a follow-up questionnaire similar to the baseline questionnaire was distributed by post to participants who completed the baseline measurement. Data collection took place in 2017 and 2018. The Medical Ethics Committee of Erasmus MC University Medical Center in Rotterdam declared that the rules lead down in the Medical Research Involving Human Subjects Act (also known by its Dutch abbreviation WMO), do not apply to this research (reference number: MEC-2016-559).

**Figure 1:**
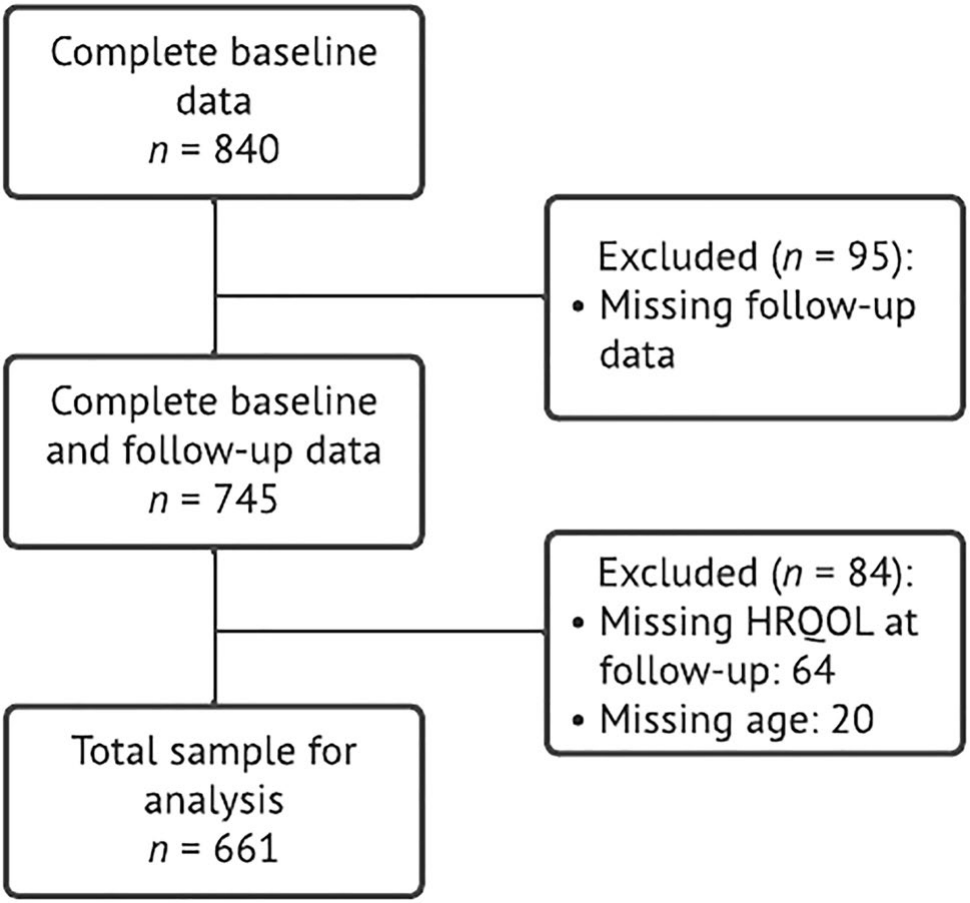
Population of analysis.

Data from 840 participants who provided informed consent and filled in the baseline questionnaire were available for this study. Participants who dropped out at follow-up (*n* = 95) were excluded. For the analysis, participants with missing data in the outcome variable (*n* = 64), age (*n* = 20), and sex (*n* = 0) were excluded, resulting in 661 (78.7%) subjects included. A flow diagram of the population of analysis is presented in Fig. 1.

### Measures

#### Physical and mental health-related quality of life (HRQOL)

The outcome measure used in this study is health-related quality of life (HRQOL). Physical and mental HRQOL were measured by the 12-item Short Form Health Survey (SF-12). Previous findings support that the SF-12 can be a reliable and valid measure to assess health-related quality of life^8,14^. The SF-12 covers eight health domains: general health, mental health, vitality, social functioning, role limitation due to physical health problems, role limitation due to emotional problems, bodily pain, and physical functioning. These domains are summarised in a Physical Component Summary (PCS) and Mental Component Summary (MCS), ranging from 0 to 100^14^. As differences between country-specific scores are minimal, it is recommended to use the standard (U.S.-derived) scoring of the SF-12 to allow for comparison and interpretation of the data across countries^8^. Prior research showed that Dutch weights resemble US weights closely^14^. Summary scores were transformed into standard scores, with a mean score of 50 and standard deviation of 10^8^. Higher scores represent higher quality of life. A change of 3 units or more in PCS and MCS is considered clinically meaningful^15^.

#### Socio-demographic factors

Socio-demographic characteristics assessed at baseline were included as covariates. Age was grouped into 65–79 years and ≥ 80 years. Household composition was categorised into living with others or living alone. Education level was split into two categories based on the International Standard Classification of Education (ISCED). ISCED level 0–5 was categorised as ‘secondary or lower’ and ISCED level 6–8 was categorised as ‘tertiary or higher’^16^.

#### Health-related factors

Baseline health indicators assessed were multi-morbidity, frailty, activities of daily living (ADL) limitations, loneliness, risk of medication-related problems, risk of malnutrition, and falls. Multi-morbidity was defined as having two or more chronic conditions^4^. The Cumulative Illness Rating Scale (CIRS) is a validated tool to measure multi-morbidity^17^. A list of 13 common chronic conditions (e.g. hypertension, stroke, diabetes) was provided^17^. Participants indicated whether they had one or more chronic condition(s) diagnosed by a physician. Frailty was measured by the 15-item Tilburg Frailty Indicator (TFI), which has been validated among Dutch community-dwelling older adults^18^. The score on overall frailty ranged from 0 to 15. Participants with a total TFI-score ≥ 5 were considered frail^18^. ADL limitations were assessed with the Groningen Activity Restriction Scale (GARS), range 18–72^19^, with higher scores representing a lower independence level. ‘Activities of daily living’ (ADL) concern routine tasks that comprise everyday living. Prior research has confirmed the validity of this scale in a community-based sample^19^. Loneliness was evaluated by the 6-item De Jong-Gierveld Loneliness Scale, a reliable and valid measurement instrument for overall, emotional and social loneliness^20^. Overall loneliness scores varied between 0–1: ‘No loneliness’, 2–4: ‘Moderately intense loneliness’, and 5–6: ‘Intense loneliness’. Overall loneliness scores were dichotomised in ‘not lonely’ (score 0–1) and ‘lonely’ (score 2–6). The risk of medication-related problems was measured by the Medication Risk Questionnaire (MRQ)^21^. The MRQ is a validated scale that can assess polypharmacy, inappropriate prescribing and poor adherence^22^. Eight items of the MRQ were summed to calculate the risk of medication-related problems^21^. The scores were dichotomised into: ‘low risk’ (score 0–3) or ‘high risk’ (score ≥ 4) of medication-related problems^22^. The risk of malnutrition was assessed with the Short Nutritional Assessment Questionnaire 65+ (SNAQ65+)^23^ and dichotomised in ‘low risk’ and ‘high risk’. The SNAQ65+ has been validated in community-dwelling older adults and can be used to determine malnutrition^23^. This study used two items of the SNAQ65+: appetite and walking stairs. The item ‘mid-upper-arm circumference’ was excluded from the score calculation as this data was not available. Instead ‘unintentional weight loss’ measured by one item of the TFI was used^24^. If a participant lost 6 kg or more during the last 6 months or 3 kg or more in the last month, this was defined as a high risk of malnutrition. Participants with poor appetite and problems with walking stairs and no weight loss, or no indications at all for malnutrition, were categorised as low risk. Falls were self-reported by asking participants “Have you had a fall in the last 12 months?”^25^. Fall status was dichotomised into has ‘fallen one or more times’ versus ‘no falls’.

#### Lifestyle factors

Physical activity, risk of alcohol harm and smoking were included as lifestyle factors. Physical activity was assessed with one item of a validated frailty instrument for primary care (SHARE-FI)^26^ to report the frequency of low to moderate-level activities, such as gardening or walking. Responses were dichotomised into ‘once a week or less’ and ‘more than once a week’. Risk of alcohol harm was assessed by three items of the Alcohol Use Disorders Identification Test (AUDIT-C), which is effective in screening high risk alcohol use among adults^27^. Scores range between 0 and 12, with 0 indicating the lowest and 12 the highest risk. The variable was dichotomised (≥ 3 in women and ≥ 4 in men) to indicate whether a person was at risk of alcohol abuse or dependence^27^. One item assessed current smoking (yes/no).

### Statistical analysis

Descriptive statistics were performed to describe participant characteristics using mean (SD) or number of participants (%) for the total study sample. Multivariate linear regression was used to assess the association between factors and HRQOL at follow-up. Regression analyses were conducted separately for the outcome variables PCS and MCS. Unstandardized regression coefficients (B) with 95% confidence intervals (95% CI) were calculated for each variable. Results were considered significant at *P* < 0.05. After excluding participants with missing data on health-related quality of life, age and sex, the proportion of missing data for each of the other measures was below 5%. Therefore, the analysis was conducted without imputation as the impact on the results of the analysis would likely be minimal^28^. To evaluate whether the association between factors and health-related quality of life was modified by socio-demographic factors (age, sex, education level, household composition), an interaction term was added to the PCS and MCS model. The interaction term socio-demographic variable*associated factor was added to the linear regression model, adjusted for all the other variables. The 2-sided significance threshold, after Bonferroni correction for multiple testing, was set at (*P* = 0.05/46 = < 0.001)^29^. To assess the correlation between the independent variables a variance inflation factor (VIF) was used by performing a multi-collinearity test. Variables are highly related when a VIF value is greater than 10^30^. Analyses were conducted using SPSS version 25.0 (IBM Corp., Armonk, NY, USA).

### Ethical approval

The Medical Ethics Committee of Erasmus MC University Medical Center in Rotterdam waived the need for approval in the study (reference number: MEC-2016-559). All procedures performed as part of the study were in accordance with the ethical standards of the institutional committee and with the 1964 Helsinki declaration and its later amendments. All participants provided written informed consent.

## Results

### Participant characteristics

The mean age of participants was 77.4 years ± 6.0 years, and 47.2% were women. Most participants had a secondary education level or lower (78.4%). Furthermore, 492 participants (74.4%) reported having two or more health conditions (i.e. multi-morbidity). Table 1 presents the characteristics of the study population at baseline.

**Table 1:** Baseline characteristics of community-dwelling older adults (*n* = 661).

Baseline variables	Value
Age (years)	77.4 ± 6.0
Sex, female	312 (47.2%)
Education level	
Secondary or lower	518 (78.4%)
Tertiary or higher	135 (20.4%)
Household composition, living alone	254 (38.4%)
Multimorbidity, yes	492 (74.4%)
Frailty, yes	190 (28.7%)
ADL (GARS; score)	23.9 ± 8.7
Loneliness, yes	238 (36.0%)
Risk of medication-related problems, yes	220 (33.3%)
Risk of malnutrition, yes	35 (5.3%)
Falls, yes	157 (23.8%)
Physical activity	
Once a week or less	173 (26.2%)
More than once a week	483 (73.1%)
Risk of alcohol harm, yes	285 (43.1%)
Smoking, yes	62 (9.4%)

*SD* standard deviation, *ADL* Activities of Daily Living, *GARS* Groningen Activities Restriction Scale.

Presented as mean ± SD or *n* (%).

Missing items: Education level = 8; Household composition = 23; Multimorbidity = 4; Frailty = 30; ADL = 5; Loneliness = 14; Medication risk = 5; Falls = 18; Physical activity = 5; Alcohol risk = 10.

### Factors associated with physical HRQOL

Table 2 presents the results of the univariate and multivariate linear regression models for the PCS of HRQOL. Interaction analyses revealed no statistically significant interactions for PCS (*P* > 0.001). For each model, the correlation between the independent variables was within acceptable limits (all VIF < 2)^30^. The multivariate model for PCS showed that women had a significantly worse PCS (B = − 2.38; 95% CI: − 3.68, − 1.07) compared to men. Furthermore, participants with multi-morbidity experienced a lower quality of life regarding physical HRQOL (B = − 2.59; 95% CI: − 4.17, − 1.00) compared to those with less than two health conditions. PCS was also significantly lower in participants indicated as frail (B = − 5.56; 95% CI: − 7.37, − 3.75) compared to non-frail participants. Moreover, the PCS decreased as the score on ADL limitations increased (B = − 0.63; 95% CI: − 0.72, − 0.53). Finally, participants at high risk for medication-related problems had a 2.84 (95% CI: − 4.28, − 1.40) lower physical HRQOL score compared to participants with a low risk of medication-related problems.

**Table 2:** Linear regression models on associations between associated factors of HRQOL and PCS at follow-up.

	Univariate model^a^		Multivariate model^b^	
	B* (95% CI)	*P*-value	B* (95% CI)	*P*-value
Age				
65–79 years	Ref		Ref	
≥ 80 years	− 6.76 (− 8.57, − 4.95)	**< 0.001**	− 0.84 (− 2.27, 0.59)	0.250
Sex				
Male	Ref		Ref	
Female	− 4.51 (− 6.25, − 2.77)	**< 0.001**	− 2.38 (− 3.68, − 1.07)	**< 0.001**
Education level				
Tertiary or higher	Ref		Ref	
Secondary or lower	− 3.37 (− 5.54, − 1.19)	**0.002**	− 1.00 (− 2.54, 0.53)	0.199
Household composition				
Living with others	Ref		Ref	
Living alone	− 5.76 (− 7.53, − 4.00)	**< 0.001**	− 0.41 (− 1.84, 1.02)	0.577
Multimorbidity				
0–1 health conditions	Ref		Ref	
≥ 2 health conditions	− 8.68 (− 10.61, − 6.75)	**< 0.001**	− 2.59 (− 4.17, − 1.00)	**0.001**
Frailty status				
Not frail	Ref		Ref	
Frail	− 14.68 (− 16.29, − 13.08)	**< 0.001**	− 5.56 (− 7.37, − 3.75)	**< 0.001**
ADL (GARS; score)	− 0.93 (− 1.00, − 0.86)	**< 0.001**	− 0.63 (− 0.72, − 0.53)	**< 0.001**
Loneliness				
Not lonely	Ref		Ref	
Lonely	− 5.79 (− 7.59, − 3.99)	**< 0.001**	1.38 (− 0.05, 2.81)	0.058
Medication-related problems				
Low risk	Ref		Ref	
High risk	− 7.68 (− 9.47, − 5.89)	**< 0.001**	− 2.84 (− 4.28, − 1.40)	**< 0.001**
Malnutrition				
Low risk	Ref		Ref	
High risk	− 10.00 (− 13.87, − 6.12)	**< 0.001**	0.15 (− 2.76, 3.06)	0.919
Falls				
No falls	Ref		Ref	
≥ 1 falls	− 7.65 (− 9.66, − 5.64)	**< 0.001**	− 0.88 (− 2.45, 0.70)	0.274
Physical activity				
More than once a week	Ref		Ref	
Once a week or less	− 8.35 (− 10.25, 6.45)	**< 0.001**	− 1.12 (− 2.68, 0.44)	0.158
Alcohol harm				
Low risk	Ref		Ref	
High risk	3.38 (1.61, 5.15)	**< 0.001**	0.57 (− 0.72, 1.86)	0.384
Smoking				
No	Ref		Ref	
Yes	− 2.36 (− 5.39, 0.67)	0.126	− 0.93 (− 3.05, 1.19)	0.391

*CI* Confidence Interval, *ADL* Activities of Daily Living, *GARS* Groningen Activities Restriction Scale.

Significant P-values (< 0.05) in bold.

*Unstandardised regression coefficient.

^a^The predictor variables were entered separately in the univariate model.

^b^The predictor variables were entered simultaneously in the multivariate model.

### Factors associated with mental HRQOL

Table 3 presents the results of the univariate and multivariate linear regression models for the MCS of HRQOL. There were no statistically significant interactions for MCS (*P* > 0.001). For each model, the correlation between the independent variables was within acceptable limits (all VIF < 2)^30^. In the univariate model, participants of 80 years and older reported lower quality of life regarding the MCS compared to younger participants (B = − 1.65; 95% CI: − 3.24, − 0.06). However, when controlling for all factors in the model, higher age was associated with a 2.07 (95% CI: 0.47, 3.68) increase in MCS. Similarly, the univariate model showed a 1.34 (95% CI: − 2.90, 0.23) reduction in MCS among participants living alone, while in the multivariate model participants living alone had a significantly higher MCS (B = 3.43; 95% CI: 1.82, 5.03) compared to participants living with others. Furthermore, participants indicated as frail reported a significantly lower quality of life regarding MCS (B = − 6.65; 95% CI: − 8.69, − 4.62) compared to non-frail participants. In addition, having a higher score on ADL limitations was significantly associated with reduced MCS (B = − 0.18; 95% CI: − 0.29, − 0.07). Finally, participants classified as lonely had a significantly lower MCS (B = − 3.64; 95% CI: − 5.25, − 2.03) compared to participants who were not at risk of loneliness.

**Table 3:** Linear regression models on associations between associated factors of HRQOL and MCS at follow-up.

	Univariate model^a^		Multivariate model^b^	
	B* (95% CI)	*P*-value	B* (95% CI)	*P*-value
Age				
65–79 years	Ref		Ref	
≥ 80 years	− 1.65 (− 3.24, − 0.06)	**0.043**	2.07 (0.47, 3.68)	**0.011**
Sex				
Male	Ref		Ref	
Female	− 1.72 (− 3.22, − 0.22)	**0.024**	− 1.16 (− 2.62, 0.31)	0.122
Education level				
Tertiary or higher	Ref		Ref	
Secondary or lower	− 1.76 (− 3.63, 0.11)	0.065	− 0.57 (− 2.29, 1.16)	0.519
Household composition				
Living with others	Ref		Ref	
Living alone	− 1.34 (− 2.90, 0.23)	0.094	3.43 (1.82, 5.03)	**< 0.001**
Multimorbidity				
0–1 health conditions	Ref		Ref	
≥ 2 health conditions	− 4.17 (− 5.87, − 2.47)	**< 0.001**	− 1.42 (− 3.20, 0.35)	0.116
Frailty status				
Not frail	Ref		Ref	
Frail	− 9.48 (− 10.97, − 8.00)	**< 0.001**	− 6.65 (− 8.69, − 4.62)	**< 0.001**
ADL (GARS; score)	− 0.44 (− 0.52, − 0.36)	**< 0.001**	− 0.18 (− 0.29, − 0.07)	**0.001**
Loneliness				
Not lonely	Ref		Ref	
Lonely	− 6.39 (− 7.88, − 4.89)	**< 0.001**	− 3.64 (− 5.25, − 2.03)	**< 0.001**
Medication-related problems				
Low risk	Ref		Ref	
High risk	− 4.95 (− 6.51, − 3.40)	**< 0.001**	− 1.32 (− 2.93, 0.30)	0.110
Malnutrition				
Low risk	Ref		Ref	
High risk	− 8.63 (− 11.91, − 5.35)	**< 0.001**	− 2.37 (− 5.65, 0.90)	0.155
Falls				
No falls	Ref		Ref	
≥ 1 falls	− 4.57 (− 6.31, − 2.82)	**< 0.001**	− 1.35 (− 3.12, 0.41)	0.133
Physical activity				
More than once a week	Ref		Ref	
Once a week or less	− 3.72 (− 5.41, − 2.04)	**< 0.001**	− 0.43 (− 2.19, 1.32)	0.627
Alcohol harm				
Low risk	Ref		Ref	
High risk	1.64 (0.12, 3.16)	**0.034**	0.48 (− 0.97, 1.92)	0.517
Smoking				
No	Ref		Ref	
Yes	− 1.67 (− 4.24, 0.90)	0.203	− 1.08 (− 3.47, 1.30)	0.373

*CI* Confidence Interval, *ADL* Activities of Daily Living, *GARS* Groningen Activities Restriction Scale.

Significant P-values (< 0.05) in bold.

*Unstandardised regression coefficient.

^a^The predictor variables were entered separately in the univariate model.

^b^The predictor variables were entered simultaneously in the multivariate model.

## Discussion

This study aimed to identify the factors associated with health-related quality of life (HRQOL) among community-dwelling older adults. Frailty and a higher score on activities of daily living (ADL) limitations were negatively associated with both physical and mental HRQOL. Female sex, multi-morbidity, and a high risk of medication-related problems were independently associated with reduced physical HRQOL, whereas loneliness was associated with reduced mental HRQOL. In contrast, higher age and living alone were associated with better mental HRQOL in the multivariate models.

Frailty was associated with reduced physical and mental HRQOL at follow-up. This is in line with previous studies^12,31–33^. Frailty is characterised by increased vulnerability and may result in weight loss, fatigue, low levels of physical activity, and depressed mood^34^. Frail older adults are at increased risk of poor health outcomes resulting from falls, disability, and hospitalisation, which may negatively impact HRQOL^31,34^. A higher score on ADL limitations was also significantly associated with a reduced Physical Component Summary (PCS) score and Mental Component Summary (MCS) score. Due to the strong relationship between a person’s ability to perform activities and the PCS score, this result was to be expected^35^. Loss of muscle strength and mobility problems, especially the ability to walk, are associated with reduced physical HRQOL^35–37^. In addition, it has been shown that loss of independence in general, and dependency regarding eating, bathing and toileting specifically, is associated with a decline in mental HRQOL^35,36^.

Consistent with previous findings, women were more likely than men to have reduced physical HRQOL^9,10,38^. A possible explanation for sex differences in HRQOL is rooted in the pattern of chronic conditions. More specifically, women are more prone to musculoskeletal diseases than men^39,40^. Musculoskeletal conditions may contribute to pain and disability, particularly in women, and are associated with worse physical HRQOL^9^. Not only the type of condition but also the number of chronic conditions may negatively impact HRQOL^41^. Consistent with previous studies, our results showed that multi-morbidity was associated with poorer physical HRQOL^39,41,42^. Furthermore, the present study confirms the high risk of medication-related problems as a predictor of low physical HRQOL^43,44^. However, no association was found with mental HRQOL in contrast to a previous study^43^. In a study by Zhang et al.^44^, lower HRQOL was associated with polypharmacy, multi-morbidity, difficulties taking medications as prescribed, and using medications with a narrow therapeutic index. Further research is recommended to clarify the association between medication-related risk factors and HRQOL.

Participants who were classified as lonely had a lower mental HRQOL compared to participants who were not at risk of loneliness. Unlike previous research, our findings did not show an association between loneliness and physical HRQOL^45,46^. A study by Tan et al.^46^ showed a stronger association between emotional loneliness and mental HRQOL compared to social loneliness, this may suggest that older adults who miss an intimate or emotional relationship are at increased risk of poor mental HRQOL. Further research is needed to explore the factors contributing to poor mental HRQOL among older adults who are lonely. Furthermore, the univariate regression model showed that higher age (≥ 80 years) was associated with reduced mental HRQOL. In contrast, higher age was associated with increased mental HRQOL in the multivariate regression model. This result was not reported in the literature^10^. Gooding et al.^47^ suggested that older-old adults (≥ 80 years) have a better-developed capacity for resilience, particularly regarding emotional regulation and problem-solving, compared to younger-old adults (65–79 years) which could explain these findings. Moreover, the univariate model showed lower mental HRQOL among participants living alone. However, when adjusted for other variables, participants living alone had a significantly higher mental HRQOL. This finding challenges a common belief that living alone negatively impacts HRQOL^48^. According to Burnette et al.^49^, those who live alone have high levels of social interaction and participation. More specifically, living alone can have positive effects on younger-old adults and those living in urban areas. Future studies need to explore if this finding holds among various age groups and settings.

A strength of our study is the comprehensive assessment of factors, including socio-demographic, health, and lifestyle factors. In addition, we were able to maintain a relatively high response rate during follow-up. However, this study also has some limitations. First, participants were recruited by sending a participation letter, which may have resulted in selection bias with underrepresentation of vulnerable participants. Lifestyle and health behavior was assessed using a self-reported questionnaire, which may cause under- or over reporting of (un)healthy behavior. Therefore, findings must be interpreted with caution. In addition, reliance on self-reported information can lead to misclassification as participants have to recall events. Objectively measured outcomes can be used to confirm our findings. Second, some variables were collapsed into dichotomous categories, which may have resulted in loss of information. Future studies are recommended to explore factors, including frailty, loneliness and malnutrition, in more detail, particularly regarding their social dimension. These factors may have a considerable effect on the association between age and HRQOL, and living alone and HRQOL. Finally, due to the limited observation time of 6 months between baseline and follow-up, a causal relationship cannot be inferred. Further research, including multiple follow-up measurements, is required to confirm the direction of the associations. Finally, the possibility of generalisation to other cultural contexts remains unclear. Future studies need to determine whether cultural factors might change the associations observed within our study.

The results of this study confirm that HRQOL is associated with multiple factors, including socio-demographic, health and lifestyle factors^10^. Longitudinal research is needed to comprehensively examine the (bi-)directional associations between factors and HRQOL over time. Future studies could assess socioeconomic status more extensively by including, for example, neighbourhood socioeconomic characteristics, socioeconomic factors earlier in life, and social support. In order to prevent morbidity in older adults, prevention strategies could focus on the role of physical activity in perceived quality of life^50^. Previous studies showed an association between maintaining a good physical condition and a better quality of life and cognitive function^50,51^. The findings of this study imply that future interventions targeting health and autonomy promotion among community-dwelling older adults could be tailored to subpopulations with relatively poor self-reported HRQOL, such as frail or lonely older adults. Additional research is needed to extend our knowledge of the factors related to HRQOL in older (pre)frail adults. This information can be useful for clinicians working with older people to identify those at risk of reduced quality of life and to target interventions accordingly.

## Conclusion

Our findings expand evidence from previous cross-sectional studies indicating an association between higher age, female sex, living alone, multi-morbidity, frailty, a higher score on activities of daily living (ADL) limitations, loneliness, a high risk of medication-related problems and HRQOL. The results of this study show the importance of socio-demographic characteristics in relation to HRQOL, encouraging a better collaboration between health and social care services. Further longitudinal research is needed to confirm our findings and understand the role of frailty in the relationship between risk factors and HRQOL.

## Data Availability

The datasets generated during and analyzed during the current study are available from the corresponding author on reasonable request.
